# No Evidence that Infection Alters Global Recombination Rate in House Mice

**DOI:** 10.1371/journal.pone.0142266

**Published:** 2015-11-09

**Authors:** Beth L. Dumont, Amy A. Devlin, Dana M. Truempy, Jennifer C. Miller, Nadia D. Singh

**Affiliations:** 1 Initiative in Biological Complexity, North Carolina State University, Raleigh, North Carolina, United States of America; 2 Department of Biological Sciences, North Carolina State University, Raleigh, North Carolina, United States of America; University of Toledo School of Medicine, UNITED STATES

## Abstract

Recombination rate is a complex trait, with genetic and environmental factors shaping observed patterns of variation. Although recent studies have begun to unravel the genetic basis of recombination rate differences between organisms, less attention has focused on the environmental determinants of crossover rates. Here, we test the effect of one ubiquitous environmental pressure–bacterial infection–on global recombination frequency in mammals. We applied MLH1 mapping to assay global crossover rates in male mice infected with the pathogenic bacterium *Borrelia burgdorferi*, the causative agent of Lyme Disease, and uninfected control animals. Despite ample statistical power to identify biologically relevant differences between infected and uninfected animals, we find no evidence for a global recombination rate response to bacterial infection. Moreover, broad-scale patterns of crossover distribution, including the number of achiasmate bivalents, are not affected by infection status. Although pathogen exposure can plastically increase recombination in some species, our findings suggest that recombination rates in house mice may be resilient to at least some forms of infection stress. This negative result motivates future experiments with alternative house mouse pathogens to evaluate the generality of this conclusion.

## Introduction

The exchange of genetic material between homologous chromosomes via crossing over is a key and defining step of the meiotic cell cycle. Crossing over is essential for proper chromosome segregation, with too few or poorly positioned crossovers posing leading risk factors for aneuploidy and infertility [1]. At the same time, crossing over (and, more broadly, recombination) is an important mechanism generating DNA diversity. Recombination controls the rate at which new haplotypes are created and influences their frequencies within populations.

In spite of its significance for the maintenance of genome integrity and evolution, there is tremendous variation for recombination rate between species [2–5], among individuals [6–14], and within single genomes [15–17]. Some of this variation is under genetic control. Classical genetic experiments have demonstrated a clear heritable component to population variation in crossover frequency [18–20]. More recently, quantitative genetic [13,21,22], association analyses [11,23–25], and candidate gene-driven approaches [23,26] have identified specific loci, including single genes, contributing to natural variation in recombination rates.

As our understanding of the genetic control of recombination rates continues to grow, it has become clear that genes do not account for all observable variation in this phenotype. In fact, narrow-sense heritability estimates indicate that most recombination rate variation cannot be explained by additive genetic factors alone [6,7,27], suggesting an important contribution from the environment. Changes in diet [28,29], temperature [30–38], age [39–43], and social stress [44] have been previously shown to elicit plastic, within generation responses in recombination rate, consistent with the possibility of a general stress-induced recombination response [45]. Recent studies have demonstrated that exposure to xenobiotic estrogens can also trigger changes in global recombination frequency in house mice [46,47], including an increase in the fraction of aneuploid gametes [46].

Despite these important contributions, there are many unanswered questions regarding the interplay of environment and recombination. For one, most studies have been restricted to *Drosophila*, plant, and house mouse model systems. Thus, the universality of these observations across the eukaryotic kingdom remains largely untested. Additionally, a limited number of environmental agents have been specifically tested for an effect on recombination. It is unclear how sensitive (or resilient) recombination rates are to the range of abiotic variables organisms encounter in their environment.

Pathogens, including infectious bacteria, are a ubiquitous feature of an organism’s environment and present a persistent source of physiological stress. Theoretical models indicate that increased genetic mixing via recombination can allow organisms to more rapidly evolve to shifting parasitic pressures in their environment, a consideration that may pose a major evolutionary advantage to sex and recombination [48–50]. Indeed, sex and meiotic recombination have been shown to evolve [51–53] and plastically increase [54–56] in response to pathogen pressures in species with facultative sexual reproduction. However, evidence that pathogen pressures can trigger increases in meiotic recombination rates in host species with obligate sexual reproduction is currently limited to *Drosophila* [57].

Here, we build on this theoretical foundation to test for an effect of bacterial infection on global meiotic crossover rates in house mice (*Mus musculus*), a species with multiple genetic modifiers [21,22,58] and established non-genetic determinants [40,46,47] of recombination rate variation. In contrast to theoretical predictions and the hypothesized link between stress and recombination [45,59], we find no evidence in support of a pathogen-associated recombination rate response in house mice. We discuss potential explanations for this negative result, as well as the biological implications of this finding.

## Methods

### Animal Husbandry and Ethics Statement

All aspects of this project were carried out in strict accordance with protocols approved by the North Carolina State University Institutional Animal Care and Use Committee (Protocols 13-095-B and 13-066-B). Throughout the course of this experiment, animal health was monitored at least once per day by trained animal technicians, veterinary staff, or one of the authors.

Mating pairs of strains C57BL/6J (B6) and PWD/PhJ (PWD) were purchased from The Jackson Laboratory and housed at the Biological Resources Facility at North Carolina State University under specific pathogen free conditions. Animals were provided with food (PicoLab^®^ Mouse Diet 20 5058*) and water *ad libitum*. Mice were purpose-bred by intra-strain crosses to generate progeny reared under controlled laboratory conditions. Male pups were weaned into same sex groups at approximately 3 weeks of age and subsequently isolated into individual cages at 8 weeks.

### Bacterial Cultures and Experimental Design

An infectious clonal isolate of *B*. *burgdorferi* (B31-MI-16) was cultured at 34C in Barbour-Stoenner-Kelly II (BSKII) medium supplemented with 6% rabbit serum [60,61]. After reaching mid-logarithmic phase (5x10^7^ bacteria/mL) the culture was diluted to a concentration of 50,000 cells/50μL for intradermal injection.

Eight-week-old adult male mice were treated with Nair™ hair removal product to expose a patch of bare skin and allowed to recover for 24 hours. Animals were then subjected to one of three alternative treatments. Mice in the first group were injected intradermally with 50,000 cells/50 μL of *B*. *burgdorferi*. The second treatment group was injected with sterile BSKII media (media control). The third group was not subjected to any further treatment (Nair-only). Animals were then aged 10–14 days under sterile conditions in a BSL-2 environment. During this time, one PWD male in the Nair-only treatment group was found dead of apparent natural causes.

Mice were sacrificed by over-exposure to isoflurane gas in a sealed container at approximately 10 weeks of age. The left testis was dissected and immediately disaggregated with a handheld blender. Testis cells were cultured in BSKII media supplemented with phosphomycin and rifampicin for 14 days, and then examined by dark field microscopy to confirm the presence of *B*. *burgdorferi*.

### Spermatoctye Spreads and Immunostaining

Spermatocyte cell spreads were made from the right testis as previously described [62] and subjected to immunostaining according to published protocols [10,22]. Slides were blocked and antibodies were diluted in 1x antibody dilution buffer [10x ADB: 2.5 mL normal donkey serum (Jackson ImmunoResearch), 22.5 mL 1x PBS, 0.75 g bovine serum albumin (Fraction V; Fisher Scientific), and 12.5 uL Triton X-100]. The following primary antibodies were used at 1:100 dilution: mouse anti-MLH1 (BD), goat anti-SCP3 (Santa Cruz Biotechnology), and human anti-centromere polyclonal (Antibodies, Inc). The following secondary antibodies were used at 1:200 dilution: donkey anti-mouse Alexa Fluor 488, donkey anti-goat Rhodamine Red-X, and donkey anti-human Coumarin AMCA (Jackson Immunoresearch). Slides were mounted in ProLongGold antifade (Promega) prior to microscopic analysis.

### Microscopy and Image Analysis

Slides were analyzed with a Leica DM5500 B microscope equipped with a Photometrics CoolSNAP HQ^2^ CCD camera and a 63x oil-immersion objective lens. Images were captured as RGB stacks in Leica Application Suite (v. 2.3.5) software and stored as high-resolution tiff files. Images were subsequently cropped and the fluorescent intensity adjusted using ImageJ software.

We aimed to capture approximately 25 well-stained late pachytene stage spermatocytes per animal. Cells in this meiotic sub-stage were defined by two key criteria: (1) the complete co-localization of SCP3 signals along the paired homologous chromosome axes and (2) a minimum of one MLH1 focus per autosome, excepting the possibility of one achiasmate bivalent per cell. Spermatocytes that appeared damaged during preparation, exhibited synaptic defects, or with bulbous chromosome termini (indicative of transition into diplotene) were not imaged. For each cell, the number of autosomal MLH1 foci was scored. Given that the dynamics of the X and Y chromosomes are temporally decoupled from those of the autosomes during early meiosis [63], MLH1 foci on the heterogametic sex chromosomes were not included in this total.

### Statistical Analyses

All statistical analyses were carried out in the R environment for statistical computing (v 2.14.1) using base packages [64]. Our MLH1 dataset consists of bounded, ordinal data that do not comply with the standard assumption of normality. Consequently, we used non-parametric Mann-Whitney U-tests to compare MLH foci counts between treatment groups.

## Results and Discussion

### *B*. *burgdorferi* invade testis tissue

We used *Borrelia burgdorferi*, the causative agent of Lyme disease, as a model bacterium to test for a plastic, within generation global recombination rate response to infection in male house mice. Rodents, including house mice, are important biological reservoirs for this bacterium and play an integral role in its lifecycle [65]. *B*. *burgdorferi* infected house mice show a well-characterized and stereotyped response to infection, with an antibody-mounted immune response initiated at approximately two weeks post-infection [66]. Prior to this time point, animals display no overt symptoms of infection, even as the bacteria multiply and infiltrate tissues distant from the site of initial infection [67–70].

One possible mechanism by which *B*. *burgdorferi* infection could induce a plastic recombination response is via direct interaction of the bacterium (or bacterial secretions) with meiotic cells. To confirm that our experimental design could detect such an effect, we first tested whether intradermal injection with *B*. *burgdorferi* resulted in bacterial invasion of the testis tissue in infected animals. Importantly, dark field microscopy indicated that all testis cell cultures initiated from uninfected control animals were sterile. In contrast, *B*. *burgdorferi* were identified in cultured testis cell extracts from all infected animals, indicating bacterial colonization of the testis. To the best of our knowledge, this is the first evidence that *B*. *burgdorferi* invade testis tissue in infected animals.

### Bacterial infection elicits no change in global crossover rates

**Fig 1:**
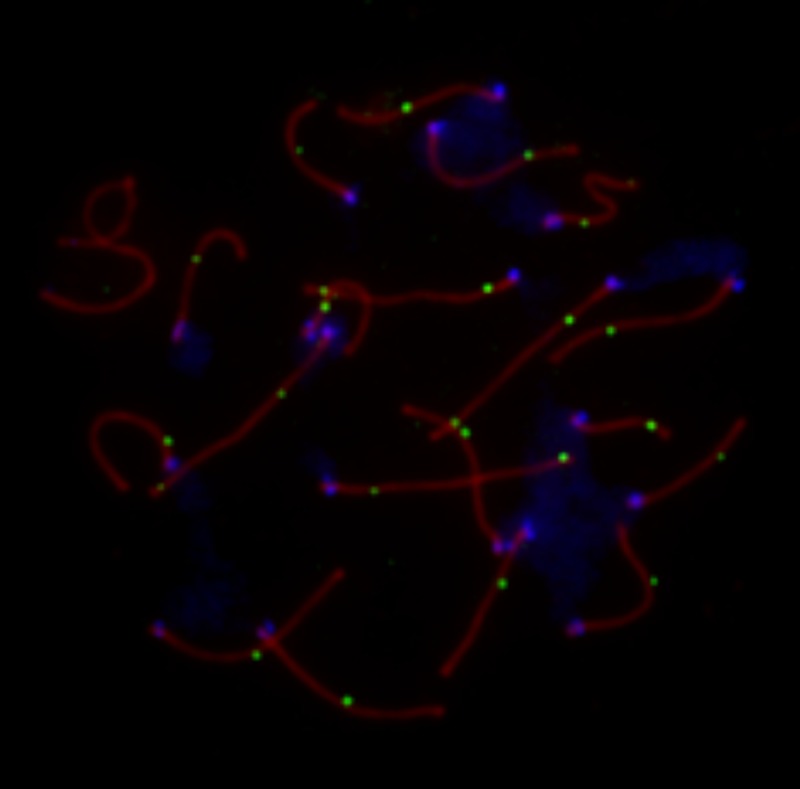
Representative image of a late pachytene spermatocyte stained with fluorescently labeled antibodies. SCP3, a component of the lateral elements of the synaptonemal complex, is stained in red. Sites of crossing over along the synaptonemal complex are denoted by green MLH1 foci. Centromeric proteins targeted by CREST antibodies are in blue. The white arrow points to the heterogametic sex chromosomes. Only MLH1 foci on autosomal bivalents were scored in this study (n = 23 for this image).

We used MLH1 mapping to estimate global crossover counts in 16 male house mice representing 2 genetically distinct inbred strains (Fig 1; Table 1). We analyzed a total of 450 spermatocytes, corresponding to an average of 28 cells per animal (range: 8–51 cells). Animals were reared under one of three experimental treatments: (1) intradermal injection with *B*. *burgdorferi*, (2) injection with sterile media, or (3) Nair-only control.

**Table 1:** MLH1 foci counts in infected and control mice.

Strain	Treatment	Animal	Number of Cells	Mean MLH1 Count	SD
B6	Infected	1	51	24.4	2.3
		2	9	23.9	2.15
		3	21	23.2	2.12
		4	29	24.5	2.03
		5	8	24.5	1.31
		6	29	24.1	2.03
		**Total**	**147**	**24.2**	**2.12**
	Nair-only	1	21	24.2	2.29
		2	26	23.8	1.77
		3	19	24.2	2.29
		**Total**	**66**	**24.0**	**2.08**
	Sterile Media	1	28	25.0	2.40
		2	39	23.6	2.25
		3	29	23.6	2.15
		4	13	23.5	2.22
		**Total**	**109**	**23.9**	**2.31**
PWD	Infected	1	44	30.1	2.49
	Nair-only	1	43	29.4	2.32
	Sterile Media	1	41	29.8	2.32

We observed a significant difference in mean MLH1 foci count between B6 and PWD mice irrespective of treatment, revealing a large difference in global crossover rate between these two genetically distinct strains (24.05 versus 29.75, respectively; Mann-Whitney U-Test *P* < 2.2x10^-16^). The numbers reported here are consistent with prior measurements in these strains [10,71] and confirm the robustness of MLH1 measurements from animals reared in distinct laboratories.

In contrast to the significant recombination rate difference between strains, we found no difference in mean MLH1 counts between treatment groups within a strain (Table 2). This result is insensitive to the control group used for comparison. Although there are minor fluctuations in mean MLH1 counts between B6 individuals within a treatment group (Table 1), these slight differences do not appear to mask an effect of infection status on global crossover frequency. One-way *ANOVA* tests performed on replicate animals within a treatment group, modeling animal replicate as a factor and the total autosomal MLH1 focus count of single spermatocytes as the response variable, are not significant *(P* > 0.05 for all tests). We conclude that *B*. *burgdorferi* infection does not alter global meiotic crossover rates in male house mice, at least at the dosage and for the exposure time considered in this experiment.

**Table 2:** *P*-values from Mann-Whitney U-Tests comparing MLH1 foci counts in infected and control animals.

	B6 Infected	PWD Infected
Nair only	0.67	0.32
Sterile Media	0.32	0.67
Both	0.36	0.41

### Determining power to find statistical differences in global crossover frequency

**Fig 2:**
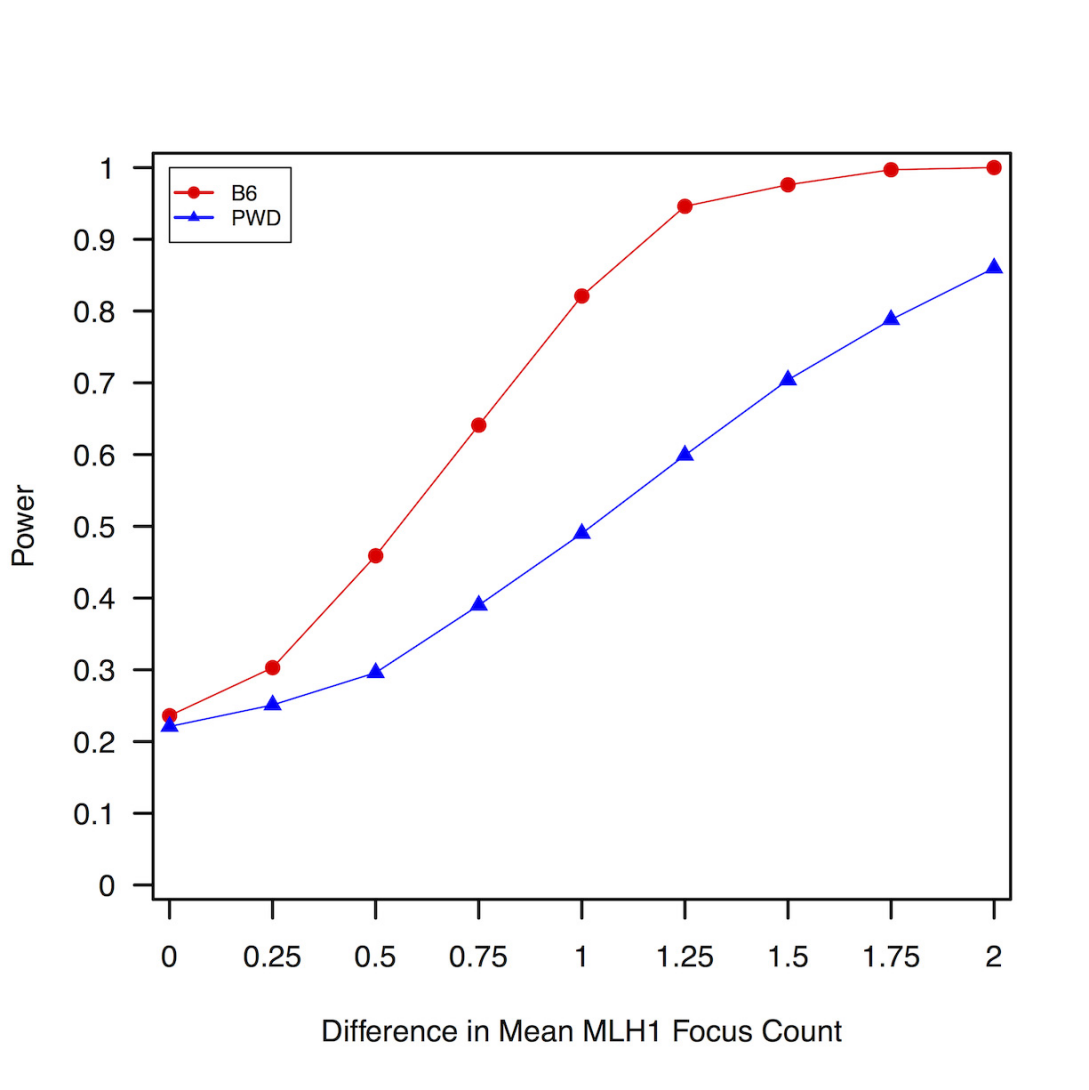
Power to find differences in mean MLH1 focus count by the Mann-Whitney U-Test. Mock MLH1 datasets for infected and uninfected animals were simulated using parameter values derived from observed MLH1 data under the assumption that focus counts are normally distributed. Power was computed as the fraction of 1000 simulated datasets with a non-parametric Mann-Whitney U-test P-value <0.05.

One interpretation for the absence of an effect of infection on global crossover frequency is a lack of statistical power to find differences between treatment groups. To calculate statistical power over a range of effect sizes, we simulated datasets that replicated the sample structure of our data, assuming that MLH1 counts follow a normal distribution with mean and standard deviation following the observed B6 and PWD values (Table 1). In reality, MLH1 count data are ordinal, but in practice, the use of random numbers sampled from a continuous distribution should have little effect on the qualitative conclusions of our simulation study. We further allowed for the possibility of inter-individual variation in average MLH1 focus counts by sampling individual values from a random normal distribution centered on the strain mean with a standard deviation of 0.5. The selection of this latter value was guided by observed differences in mean MLH1 counts between replicate animals (Table 1). With our B6 sample structure, there is excellent statistical power to detect a mean difference of ≥1 MLH1 focus (Power > 0.8 at α = 0.05 using a Mann-Whitney U-Test; Fig 2). Our power to detect differences in mean MLH1 foci counts for PWD individuals is reduced owing to a single biological replicate per treatment. However, our analysis is still well powered to find differences ≥1.5 MLH1 foci for sample sizes mirroring the collected data (Power > 0.7 at α = 0.05; Fig 2).

For both the B6 and PWD strains, approximately 20% of simulations uncover a significant difference between treatment groups in the absence of a simulated effect. These false positives are explained by chance differences between treatments that arise from random sampling of individual means.

### Infection induces no changes in the distribution of meiotic crossovers

Although there is no detectable difference in global MLH1 frequency between infected and uninfected animals of either tested strain background, there may be shifts in the distribution of crossover events, including differences in the frequency of achiasmate chromosomes or chromosomes bearing multiple MLH1 foci, that are not captured by this overall measure. We tested for a significant infection-driven response in the number of achiasmate bivalents, the number of bivalents with a single MLH1 focus, and the number of bivalents with two MLH1 foci. None of these meiotic sub-phenotypes was plastically altered in infected animals (Mann-Whitney U-Test *P* > 0.15 in all comparisons; Table 3). Similarly, the cell-to-cell variance in mean MLH1 foci number is independent of infection status (Table 3; Fligner-Killeen Test *P* > 0.35 for both strains). Although these meiotic phenotypes differ between B6 and PWD, infection status has no discernable impact on any of these sub-phenotypes.

**Table 3:** Variation in meiotic and recombination sub-phenotypes across treatment groups.

	C57BL/6J			PWD/PhJ		
	Infected	Sterile Media	Nair Only	Infected	Sterile Media	Nair Only
Average number of achiasmate bivalents per cell	0.18	0.26	0.18	0.14	0.10	0.19
Average number of bivalents with 1 MLH1 focus per cell	13.50	13.60	13.64	7.98	8.15	8.30
Average number of bivalents with 2 MLH1 foci per cell	5.30	5.11	5.17	10.71	10.66	10.44
Variance in MLH1 count	4.51	5.33	4.32	6.22	5.39	5.39
Fraction of cells with paired XY	0.94	0.92	0.91	1	0.98	1
Fraction of cells with an MLH1 focus on XY	0.29	0.34	0.27	0.32	0.24	0.28

The heterogametic X and Y sex chromosomes pair and undergo recombination across a narrow region of homology on their telomere-proximal end. Although the dynamics of pairing and recombination in this pseudoautosomal region are de-coupled from the activities of the autosomes at meiosis [63], the fraction of paired and recombining sex chromosomes at late-pachytene provides a snapshot of the temporal progression of these processes. Recent studies have highlighted several unique aspects of sex chromosome behavior during meiosis [63,72], raising the distinct possibility that infection stress could impose unique pressures on the meiotic behavior of the X and Y, independent of the autosomes. However, as above, we find no difference in the frequency of paired XY chromosomes or XY chromosomes with an MLH1 focus in the paired pseudoautosomal region at late pachytene between infected and uninfected animals (Mann-Whitney U-Test *P* > 0.4 in all tests; Table 3).

### Reconciling current observations with previous work

Despite considerable evidence for a plastic recombination rate response to diverse environmental stressors [28–45,57], here we have shown that exposure of adult male house mice to the pathogen *B*. *burgdorferi* does not alter global crossover rates or gross features of crossover distribution. Below, we summarize several distinguishing features of our study that could account for the apparent discrepancy between our findings and previous work.

First, although certain environmental variables have direct effects on recombination frequency in house mice [44,46,47], it is possible that other factors, including infection, could impact recombination rate by fundamentally distinct mechanisms. For example, *D*. *melanogaster* exhibit a strong pathogen-associated recombination response, but the observed plastic increase in recombination rates is driven, at least in part, by non-Mendelian transmission of recombinant chromatids [57]. Notably, our current study is not designed to detect differences in fertilization efficiency, differences in sperm viability, zygotic viability differences between recombinant and non-recombinant gametes, or asymmetries in meiosis that could distort transmission and lead to biological increases in recombination rate.

Second, our analysis tested the effect of exposure to one infectious pathogen, *B*. *burgdorferi*, on global crossover rates. A recombinational response to bacterial infection in house mice could be pathogen-specific, and potentially related to the nature of the mounted immune response. Although *B*. *burgdorferi* are present in the testis tissue of infected mice, the bacterium is only mildly morbific to most inbred mouse strains, producing no chronic, outward signs of disease [67–70]. Thus, *B*. *burgdorferi* infection alone may not impose sufficient stress to elicit a change in the frequency of recombination. Consistent with this possibility, exposure to heat-killed bacteria does not induce a recombinational response in *D*. *melanogaster*, even though the immune system is activated [73], whereas infection with live bacteria significantly increases recombination rates [57]. Future experiments that test for a recombination response to more virulent pathogens could yield results distinct from our findings.

Third, our study also lacks power to find small changes in global crossover rate that may arise as a result of infection (Fig 2). However, documented changes in recombination frequency in response to pathogen infection in *Drosophila* are sizeable, corresponding to ~3% change in the estimated recombination fraction, or a ~15–20% relative increase in recombination rate [57]. Similarly, exposure to environmentally relevant doses of BPA induces differences of >1 MLH1 focus in mice [46,47]. A plastic phenotypic response of these magnitudes could be reliably detected with our sample size (Fig 2).

Finally, our analysis relies on the immunodetection of MLH1 foci in late pachytene spermatocytes to estimate global crossover rates in males. Previous studies have established the accuracy and power of this immunofluorescence approach for approximating global crossover rates in single animals [10,22,74]. However, a minor subset of crossovers (<10%) is resolved by an MLH1-independent pathway in house mice [75]; any effect of infection limited to crossovers resolved via this alternative pathway would obviously go undetected by our study. In addition, the MLH1 immunofluorescence assay lacks the resolution to identify compensatory finer-scale changes in crossover rates. Finally, owing to the challenge of obtaining early meiotic cells in females [e.g., [76]], we did not address the possibility of a female-specific response to *B*. *burgdorferi* infection in the current experiment. These limitations present clear opportunities for future investigations.

### Evolutionary implications of the absence of a plastic recombination rate response

The Red Queen theory for the evolution and maintenance of sex posits that increased recombination may be selectively favored to allow species to rapidly adapt to changing parasitic pressures in their environment [77,78]. Consistent with the predictions of this hypothesis, species with both sexual and asexual modes of reproduction can shift the relative population ratio of sexual versus asexual individuals to favor sex when confronted by pathogens [51,53]. Similarly, recombination rates can plastically increase in response to infection in species with both obligate [57] and facultative sexual reproduction [59,79].

House mice are exposed to a barrage of pathogens in their wild habitats [80–82], conditions that set the stage for potential host-pathogen arms races. Despite this clear ecological opportunity, our data do not suggest that house mice attempt to genetically outwit one common bacterium in their environment (*B*. *burgdorferi*) via an increased rate of global meiotic recombination. It is possible that a pathogen-driven plastic recombination response is a biological mechanism present in only some taxa. Indeed, mathematical models have demonstrated that the conditions under which plastic recombination can evolve in diploid organisms are quite restrictive [83]. Moreover, for mammalian species with small effective population sizes, the magnitude of the selective advantage associated with moderate plastic changes in recombination rates may not be sufficient to overwhelm the power of random genetic drift. However, as is the case in *Drosophila* [57], an infection-driven recombination response in house mice could be mediated by transmission distortion rather than an overt increase in recombination rate. Future work will be aimed at specifically testing this hypothesis.

Increased recombination–whether via increased sexual reproduction, increased recombination rates, or biased transmission of recombinant gametes–is one effective strategy for evading parasites, but additional biological mechanisms are integral to host defense. In particular, mammals possess a sophisticated immune system with both innate and adaptive components. The mammalian adaptive immune system may provide an effective biological barrier to rapidly evolving biotic stressors, potentially mitigating the effect of a plastic recombination response. Indeed, plastic recombination responses have, to date, only been documented in invertebrates with comparatively simple innate immune systems. However, without first ruling out transmission distortion as a potential mechanism for pathogen-associated increased recombination in house mice and in the absence of data from other mammalian species, this possible explanation remains speculative. Vertebrates, including mammals, also have higher per base mutation rates than invertebrates [84]. The higher rate of input of new variants may effectively match the challenge of adapting to new parasitic pressures, independently of changes in recombination rate. Clearly, investigations that explicitly test predictions of the Red Queen hypothesis in diverse organisms, including other mammals, are needed.

Our findings prompt us to speculate that the relative contributions of genetic and non-genetic factors to recombination rate variation may differ between species. Although heritability estimates are not strictly comparable between studies, it is noteworthy that estimates from house mice [7,22] are consistently higher than those reported in other species [19,27,42,85]. Thus, relative to other taxa, recombination rate variation in house mice may be more strongly tied to the effects of segregating variation in recombination modifying genes and more weakly influenced by environment. If true, this possibility would add an additional layer of complexity to our nascent understanding of the mechanisms contributing to recombination rate variation.

## Supporting Information

S1 Table Per cell MLH1 counts and meiotic sub-phenotype data. (XLSX)

## References

[1] Hassold T, Hunt P. To err (meiotically) is human: the genesis of human aneuploidy. Nat Rev Genet. 2001;2: 280–291. 11283700 10.1038/35066065

[2] Coop G, Przeworski M. An evolutionary view of human recombination. Nat Rev Genet. 2007;8: 23–34. 17146469 10.1038/nrg1947

[3] Dumont BL, Payseur BA. Evolution of the genomic rate of recombination in mammals. Evolution. 2008;62: 276–294. 18067567 10.1111/j.1558-5646.2007.00278.x

[4] Smukowski CS, Noor MAF. Recombination rate variation in closely related species. Heredity. 2011;107: 496–508. 10.1038/hdy.2011.44 21673743 PMC3242630

[5] Winckler W, Myers SR, Richter DJ, Onofrio RC, McDonald GJ, Bontrop RE, et al Comparison of fine-scale recombination rates in humans and chimpanzees. Science. 2005;308: 107–111. 15705809 10.1126/science.1105322

[6] Coop G, Wen X, Ober C, Pritchard JK, Przeworski M. High-resolution mapping of crossovers reveals extensive variation in fine-scale recombination patterns among humans. Science. 2008;319: 1395–1398. 10.1126/science.1151851 18239090

[7] Dumont BL, Broman KW, Payseur BA. Variation in genomic recombination rates among heterogeneous stock mice. Genetics. 2009;182: 1345–1349. 10.1534/genetics.109.105114 19535547 PMC2728871

[8] Broman KW, Murray JC, Sheffield VC, White RL, Weber JL. Comprehensive human genetic maps: individual and sex-specific variation in recombination. Am J Hum Genet. 1998;63: 861–869. 9718341 10.1086/302011 PMC1377399

[9] Sun F, Oliver-Bonet M, Liehr T, Starke H, Ko E, Rademaker A, et al Human male recombination maps for individual chromosomes. Am J Hum Genet. 2004;74: 521–31. 14973780 10.1086/382138 PMC1182265

[10] Koehler KE, Cherry JP, Lynn A, Hunt PA, Hassold TJ. Genetic control of mammalian meiotic recombination. I. Variation in exchange frequencies among males from inbred mouse strains. Genetics. 2002;162: 297–306. 12242241 10.1093/genetics/162.1.297 PMC1462263

[11] Kong A, Thorleifsson G, Stefansson H, Masson G, Helgason A, Gudbjartsson DF, et al Sequence variants in the RNF212 gene associate with genome-wide recombination rate. Science. 2008;319: 1398–1401. 10.1126/science.1152422 18239089

[12] Borodin PM, Karamysheva TV, Belonogova NM, Torgasheva AA, Rubtsov NB, Searle JB. Recombination map of the common shrew, Sorex araneus (Eulipotyphla, Mammalia). Genetics. 2008;178: 621–632. 10.1534/genetics.107.079665 18245365 PMC2248351

[13] Thomsen H, Reinsch N, Xu N, Bennewitz J, Looft C, Grupe S, et al A whole genome scan for differences in recombination rates among three Bos taurus breeds. Mamm Genome. 2001;12: 724–728. 11641721 10.1007/s00335-001-2068-0

[14] Webb AJ, Berg IL, Jeffreys A. Sperm cross-over activity in regions of the human genome showing extreme breakdown of marker association. Proc Natl Acad Sci. 2008;105: 10471–10476. 10.1073/pnas.0804933105 18650392 PMC2483235

[15] Auton A, Fledel-Alon A, Pfeifer S, Venn O, Segurel L, Street T, et al A fine-scale chimpanzee genetic map from population sequencing. Science. 2012;336: 193–198. 10.1126/science.1216872 22422862 PMC3532813

[16] Stevison L, Noor MF. Genetic and evolutionary correlates of fine-scale recombination rate variation in Drosophila persimilis. J Mol Evol. 2010;71: 332–345. 10.1007/s00239-010-9388-1 20890595

[17] Myers S, Bottolo L, Freeman C, McVean G, Donnelly P. A fine-scale map of recombination rates and hotspots across the human genome. Science. 2005;310: 321–324. 16224025 10.1126/science.1117196

[18] Chinnici JP. Modification of recombination frequency in Drosophila. II. The polygenic control of crossing over. Genetics. 1971;69: 85–96. 5002415 10.1093/genetics/69.1.85 PMC1212691

[19] Kidwell MG. Genetic change of recombination value in Drosophila melanogaster. I. Artificial selection for high and low recombination and some properties of recombination-modifying genes. Genetics. 1972;70: 419–432. 4623519 10.1093/genetics/70.3.419 PMC1212746

[20] Charlesworth B, Charlesworth D. Genetic variation in recombination in Drosophila. I. Responses to selection and preliminary genetic analysis. Heredity. 1985;54: 71–83.

[21] Murdoch B, Owen N, Shirley S, Crumb S, Broman KW, Hassold T. Multiple loci contribute to genome-wide recombination levels in male mice. Mamm Genome. 2010;21: 550–555. 10.1007/s00335-010-9303-5 21113599 PMC3002158

[22] Dumont B, Payseur B. Genetic analysis of genome-scale recombination rate evolution in house mice. PLoS Genet. 2011;7: e1002116 10.1371/journal.pgen.1002116 21695226 PMC3111479

[23] Baudat F, Buard J, Grey C, Fledel-Alon A, Ober C, Przeworski M, et al PRDM9 is a major determinant of meiotic recombination hotspots in humans and mice. Science. 2010;327: 836–840. 10.1126/science.1183439 20044539 PMC4295902

[24] Chowdhury R, Bois PRJ, Feingold E, Sherman SL, Cheung VG. Genetic analysis of variation in human meiotic recombination. PLoS Genet. 2009;5: e1000648 10.1371/journal.pgen.1000648 19763160 PMC2730532

[25] Stefansson H, Helgason A, Thorleifsson G, Steinthorsdottir V, Masson G, Barnard J, et al A common inversion under selection in Europeans. Nat Genet. 2005;37: 129–137. 15654335 10.1038/ng1508

[26] Myers S, Bowden R, Tumian A, Bontrop RE, Freeman C, MacFie TS, et al Drive against hotspot motifs in primates implicates the PRDM9 gene in meiotic recombination. Science. 2010;327: 876–879. 10.1126/science.1182363 20044541 PMC3828505

[27] Fledel-Alon A, Leffler EM, Guan Y, Stephens M, Coop G, Przeworski M. Variation in human recombination rates and its genetic determinants. PLoS One. 2011;6: e20321 10.1371/journal.pone.0020321 21698098 PMC3117798

[28] Neel J V. A Relation between larval nutrition and the frequency of crossing over in the third chromosome of Drosophila melanogaster. Genetics. 1941;26: 506–516. 17247020 10.1093/genetics/26.5.506 PMC1209143

[29] Abdullah MFF, Borts RH. Meiotic recombination frequencies are affected by nutritional states in Saccharomyces cerevisiae. Proc Natl Acad Sci. 2001;98: 14524–14529. 11724920 10.1073/pnas.201529598 PMC64715

[30] Stern C. An effect of temperature and age on crossing-over in the first chromosome of Drosophila melanogaster. Proc Natl Acad Sci. 1926;12: 530–532. 16587124 10.1073/pnas.12.8.530 PMC1084661

[31] Plough HH. The effect of temperature on crossing over. J Exp Zool. 1917;24: 147–209.

[32] Plough HH. Further studies on the effect of temperature on crossing over. J Exp Zool. 1921;32: 187–202.

[33] Smith HF. Influence of temperature on crossing-over in Drosophila. Nature. 1936;138: 329–330.

[34] Phillips D, Jenkins G, Macaulay M, Nibau C, Wnetrzak J, Fallding D, et al The effect of temperature on the male and female recombination landscape of barley. New Phytol. 2015;208: 421–429. 10.1111/nph.13548 26255865

[35] Dowrick G. The influence of temperature on meiosis. Heredity. 1957;11: 37–49.

[36] Henderson SA, Buss ME. The superimposition of heat-induced chiasma frequency changes in Locusta migratoria. Heredity. 1989;62: 77–84. 2732090 10.1038/hdy.1989.10

[37] Henderson SA. Four effects of elevated temperature on chiasma formation in the locust Schistocerca gregaria. Heredity. 1988;60: 387–401.

[38] Powell JB, Nilan RA. Influence of temperature on crossing over in an inversion heterozygote in barley. Crop Sci. 1963;3: 11–13.

[39] Bridges CB. The relation of the age of the female to crossing over in the third chromosome of Drosophila melanogaster. J Gen Physiol. 1927;8: 689–700. 19872223 10.1085/jgp.8.6.689 PMC2140810

[40] Henderson SA, Edwards RG. Chiasma frequency and maternal age in mammals. Nature. 1968; 218: 22–28. 4230650 10.1038/218022a0

[41] Hussin J, Roy-Gagnon M-H, Gendron R, Andelfinger G, Awadalla P. Age-dependent recombination rates in human pedigrees. PLoS Genet. 2011;7: e1002251 10.1371/journal.pgen.1002251 21912527 PMC3164683

[42] Kong A, Barnard J, Gudbjartsson DF, Thorleifsson G, Jonsdottir G, Sigurdardottir S, et al Recombination rate and reproductive success in humans. Nat Genet. 2004;36: 1203–1206. 15467721 10.1038/ng1445

[43] Tedman-Aucoin K, Agrawal AF. The effect of deleterious mutations and age on recombination in Drosophila melanogaster. Evolution. 2012;66: 575–585. 10.1111/j.1558-5646.2011.01450.x 22276549

[44] Belyaev D, Borodin PM. The influence of stress on variation and its role in evolution. Biol Zent Bl. 1982;101: 705–714.

[45] Parsons PA. Evolutionary rates–effects of stress upon recombination. Biol J Linn Soc. 1988;35: 49–68.

[46] Susiarjo M, Hassold TJ, Freeman E, Hunt PA. Bisphenol A exposure in utero disrupts early oogenesis in the mouse. PLoS Genet. 2007;3: e5 17222059 10.1371/journal.pgen.0030005 PMC1781485

[47] Vrooman LA, Oatley JM, Griswold JE, Hassold TJ, Hunt PA. Estrogenic exposure alters the spermatogonial stem cells in the developing testis, permanently reducing crossover levels in the adult. PLOS Genet. 2015;11: e1004949 10.1371/journal.pgen.1004949 25615633 PMC4304829

[48] Peters AD, Lively CM. The Red Queen and fluctuating epistasis: A population genetic analysis of antagonistic coevolution. Am Nat. 1999;154: 393–405. 10523486 10.1086/303247

[49] Schmid-Hempel P, Jokela J. Socially structured populations and evolution of recombination. Am Nat. 2002;160: 403–408. 10.1086/341517 18707448

[50] Hamilton WD, Axelrod R, Tanese R. Sexual reproduction as an adaptation to resist parasites. Proc Natl Acad Sci. 1990;87: 3566–3573. 2185476 10.1073/pnas.87.9.3566 PMC53943

[51] Morran LT, Schmidt OG, Gelarden IA, Parrish RC, Lively CM. Running with the Red Queen: host-parasite coevolution selects for biparental sex. Science. 2011;333: 216–218. 10.1126/science.1206360 21737739 PMC3402160

[52] Busch JW, Neiman M, Koslow JM. Evidence for maintenance of sex by pathogens in plants. Evolution. 2004;58: 2584–2590. 15612300 10.1111/j.0014-3820.2004.tb00886.x

[53] Jokela J, Dybdahl MF, Lively CM. The maintenance of sex, clonal dynamics, and host-parasite coevolution in a mixed population of sexual and asexual snails. Am Nat. 2009;174 Suppl: S43–S53. 19441961 10.1086/599080

[54] Soper DM, King KC, Vergara D, Lively CM. Exposure to parasites increases promiscuity in a freshwater snail. Biol Lett. 2014;10: 20131091 10.1098/rsbl.2013.1091 24759366 PMC4013694

[55] Mostowy R, Engelstädter J. Host-parasite coevolution induces selection for condition-dependent sex. J Evol Biol. 2012;25: 2033–2046. 10.1111/j.1420-9101.2012.02584.x 22913382

[56] Andronic L. Viruses as triggers of DNA rearrangements in host plants. Can J Plant Sci. 2012;92: 1083–1091.

[57] Singh ND, Criscoe DR, Skolfield S, Kohl KP, Keebaugh ES, Schlenke TA. Fruit flies diversify their offspring in response to parasite infection. Science. 2015;349: 747–750. 10.1126/science.aab1768 26273057

[58] Parvanov ED, Petkov PM, Paigen K. Prdm9 controls activation of mammalian recombination hotspots. Science. 2010;327: 835 10.1126/science.1181495 20044538 PMC2821451

[59] Song J, Bent AF. Microbial pathogens trigger host DNA double-strand breaks whose abundance is reduced by plant defense responses. PLoS Pathog. 2014;10: e1004226. 10.1371/journal.ppat.1004030 PMC3974866 24699527

[60] Miller JC, von Lackum K, Babb K, McAlister JD, Stevenson B. Temporal analysis of Borrelia burgdorferi Erp protein expression throughout the mammal-tick infectious cycle. Infect Immun. 2003;71: 6943–6952. 14638783 10.1128/IAI.71.12.6943-6952.2003 PMC308935

[61] Zückert WR. Laboratory maintenance of Borrelia burgdorferi. Curr Prot in Microbiol. 2005;4:C:12C.1:12C.1.1-12C.1.10. 10.1002/9780471729259.mc12c01s4 18770608

[62] Peters AH, Plug AW, van Vugt MJ, de Boer P. A drying-down technique for the spreading of mammalian meiocytes from the male and female germline. Chromosome Res. 1997;5: 66–68. 9088645 10.1023/a:1018445520117

[63] Kauppi L, Barchi M, Baudat F, Romanienko PJ, Keeney S, Jasin M. Distinct properties of the XY pseudoautosomal region crucial for male meiosis. Science. 2011;331: 916–920. 10.1126/science.1195774 21330546 PMC3151169

[64] R Development Core Team. R: A language and environment for statistical computing 2008 R Foundation for Statistical Computing, Vienna, Austria. ISBN 3-900051-07-0.

[65] Radolf JD, Caimano MJ, Stevenson B, Hu LT. Of ticks, mice and men: understanding the dual-host lifestyle of Lyme disease spirochaetes. Nat Rev Micro. 2012;10: 87–99. 10.1038/nrmicro2714 PMC3313462 22230951

[66] Hastey CJ, Elsner RA, Barthold SW, Baumgarth N. Delays and diversions mark the development of B cell responses to Borrelia burgdorferi infection. J Immunol. 2012;188: 5612–5622. 10.4049/jimmunol.1103735 22547698 PMC3358496

[67] Barthold SW, de Souza M, Fikrig E, Persing DH. Lyme borreliosis in the laboratory mouse In: Schutzer SE, editor. Lyme Disease: Molecular and Immunologic Approaches. Plainview: Cold Spring Harbor Laboratory Press; 1992 pp. 223–242.

[68] Barthold SW, Persing DH, Armstrong AL, Peeples RA. Kinetics of Borrelia burgdorferi dissemination and evolution of disease after intradermal inoculation of mice. Am J Pathol. 1991;139: 263–273. 1867318 PMC1886084

[69] Barthold SW, Beck DS, Hansen GM, Terwilliger GA, Moody KD. Lyme borreliosis in selected strains and ages of laboratory mice. J Infect Dis. 1990;162: 133–138. 2141344 10.1093/infdis/162.1.133

[70] Ma Y, Seiler KP, Eichwald EJ, Weis JH, Teuscher C, Weis JJ. Distinct characteristics of resistance to Borrelia burgdorferi induced arthritis in C57BL/6N mice. Infect Immun. 1998;66: 161–168. 9423853 10.1128/iai.66.1.161-168.1998 PMC107872

[71] Dumont BL, Payseur BA. Evolution of the genomic recombination rate in murid rodents. Genetics. 2011;187: 643–657. 10.1534/genetics.110.123851 21149647 PMC3063662

[72] Brick K, Smagulova F, Khil P, Camerini-Otero RD, Petukhova GV. Genetic recombination is directed away from functional genomic elements in mice. Nature. 2012;485: 642–645. 10.1038/nature11089 22660327 PMC3367396

[73] Fukuyama H, Verdier Y, Guan Y, Makino-Okamura C, Shilova V, Liu X, et al Landscape of protein-protein interactions in Drosophila immune deficiency signaling during bacterial challenge. Proc Natl Acad Sci. 2013;110: 10717–10722. 10.1073/pnas.1304380110 23749869 PMC3696746

[74] Anderson LK, Reeves A, Webb LM, Ashley T. Distribution of crossing over on mouse synaptonemal complexes using immunofluorescent localization of MLH1 protein. Genetics. 1999;151: 1569–1579. 10101178 10.1093/genetics/151.4.1569 PMC1460565

[75] Holloway JK, Booth J, Edelmann W, McGowan CH, Cohen PE. MUS81 generates a subset of MLH1-MLH3-independent crossovers in mammalian meiosis. PLoS Genet. 2008;4: e1000186 10.1371/journal.pgen.1000186 18787696 PMC2525838

[76] Cheng EY, Hunt PA, Naluai-Cecchini TA, Fligner CL, Fujimoto VY, Pasternack TL, et al Meiotic recombination in human oocytes. PLoS Genet. 2009;5: e1000661 10.1371/journal.pgen.1000661 19763179 PMC2735652

[77] Jaenlke J. A hypothesis to account for the maintenance of sex within populations. Evol Theory. 1978;94: 191–194.

[78] Hamilton WD. Sex versus non-sex versus parasite. Oikos. 1980;35: 282–290.

[79] Lucht JM, Mauch-Mani B, Steiner H-Y, Metraux J-P, Ryals J, Hohn B. Pathogen stress increases somatic recombination frequency in Arabidopsis. Nat Genet. 2002;30: 311–314. 11836502 10.1038/ng846

[80] Becker SD, Bennett M, Stewart JP, Hurst JL. Serological survey of virus infection among wild house mice (Mus domesticus) in the UK. Lab Anim. 2007;41: 229–238. 17430622 10.1258/002367707780378203

[81] Moro D, Lawson MA, Hobbs RP, Thompson RCA. Pathogens of house mice on arid Boullanger Island and subantarctic Macquarie Island, Australia. J Wildl Dis. 2003;39: 762–771. 14733270 10.7589/0090-3558-39.4.762

[82] Sage RD, Heyneman D, Lim K-C, Wilson AC. Wormy mice in a hybrid zone. Nature. 1986;324: 60–63. 12356091 10.1038/324060a0

[83] Agrawal AF, Hadany L, Otto SP. The evolution of plastic recombination. Genetics. 2005;171: 803–812. 16020791 10.1534/genetics.105.041301 PMC1456799

[84] Lynch M. The origins of genome architecture Sunderland, MA: Sinauer Associates, Inc; 2007.

[85] Sandor C, Li W, Coppieters W, Druet T, Charlier C, Georges M. Genetic variants in REC8, RNF212, and PRDM9 influence male recombination in cattle. PLoS Genet. 2012;8: e1002854 10.1371/journal.pgen.1002854 22844258 PMC3406008

